# Internal Illumination to Overcome the Cell Density Limitation in the Scale‐up of Whole‐Cell Photobiocatalysis

**DOI:** 10.1002/cssc.202100832

**Published:** 2021-07-06

**Authors:** Markus Hobisch, Jelena Spasic, Lenny Malihan‐Yap, Giovanni Davide Barone, Kathrin Castiglione, Paula Tamagnini, Selin Kara, Robert Kourist

**Affiliations:** ^1^ Department of Biological and Chemical Engineering Biocatalysis and Bioprocessing Group Aarhus University Gustav Wieds Vej 10 8000 Aarhus Denmark; ^2^ Institute of Molecular Biotechnology Graz University of Technology NAWI Graz BioTechMed Petersgasse 14 8010 Graz Austria; ^3^ i3S – Instituto de Investigação e Inovação em Saúde Universidade do Porto & IBMC – Instituto de Biologia Molecular e Celular R. Alfredo Allen 208 4200-135 Porto Portugal; ^4^ Departamento de Biologia Faculdade de Ciências Universidade do Porto Rua do Campo Alegre, Edifício FC4 4169-007 Porto Portugal; ^5^ Institute of Bioprocess Engineering Department of Chemical and Bioengineering Friedrich-Alexander-Universität Erlangen-Nürnberg Paul-Gordan-Straße 3 91052 Erlangen Germany

**Keywords:** asymmetric catalysis, biocatalysis, cyanobacteria, ene-reduction, photocatalysis

## Abstract

Cyanobacteria have the capacity to use photosynthesis to fuel their metabolism, which makes them highly promising production systems for the sustainable production of chemicals. Yet, their dependency on visible light limits the cell‐density, which is a challenge for the scale‐up. Here, it was shown with the example of a light‐dependent biotransformation that internal illumination in a bubble column reactor equipped with wireless light emitters (WLEs) could overcome this limitation. Cells of the cyanobacterium *Synechocystis* sp. PCC 6803 expressing the gene of the ene‐reductase YqjM were used for the reduction of 2‐methylmaleimide to (*R*)‐2‐methylsuccinimide with high optical purity (>99 % *ee*). Compared to external source of light, illumination by floating wireless light emitters allowed a more than two‐fold rate increase. Under optimized conditions, product formation rates up to 3.7 mm h^−1^ and specific activities of up to 65.5 U g_DCW_^−1^ were obtained, allowing the reduction of 40 mm 2‐methylmaleimide with 650 mg isolated enantiopure product (73 % yield). The results demonstrate the principle of internal illumination as a means to overcome the intrinsic cell density limitation of cyanobacterial biotransformations, obtaining high reaction rates in a scalable photobioreactor.

## Graphical abstract

**Visibly green**: While light‐driven biocatalysis hosted in cyanobacteria promises a sustainable way to fine chemicals, cell density limitation remains a challenge. This limitation is overcome using internal illumination in a scalable bubble column at 200 mL reaction volume with 650 mg enantiopure 2‐methylsuccinimide (73 % isolated yield).

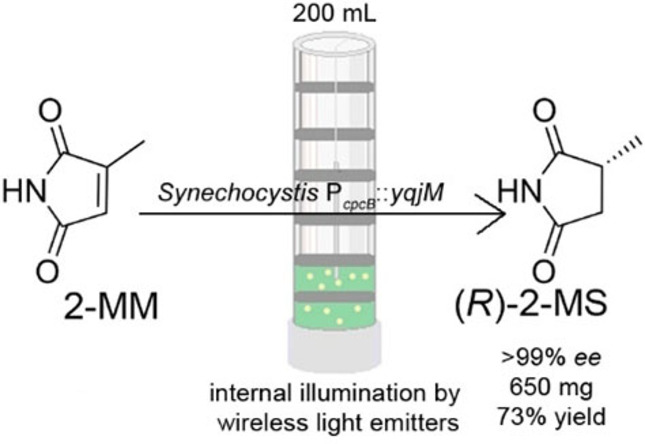

## Introduction

Photobiocatalysis has emerged as a new, exciting research area that combines photocatalysis and biocatalysis, two of the most research‐intensive fields of catalysis.^[1]^ In particular, whole‐cell biotransformations in cyanobacteria allow to produce highly selective biocatalysts from carbon dioxide and water, and to exploit photosynthesis for the regeneration of adenosine triphosphate (ATP) and redox cofactors.^[2]^ This allows the saving of stoichiometric addition of auxiliary cosubstrates such as isopropanol and glucose for redox biotransformations. As only a minor part of the donor molecule's electrons is used for the redox reaction, the use of catalytic water splitting leads to a more favorable atom economy, one of the 12 principles of green chemistry.^[3]^ Furthermore, the formation of coupled by‐products often complicates downstream processing. Using water as a sacrificial electron donor is a radical approach that has the potential to solve this critical limitation of the sustainability of enzymatic redox processes. Coupling of oxidoreductases to the photosynthetic electron transport chain of cyanobacteria has been demonstrated for a wide range of biocatalytic reactions,[^1^, ^4^] including ene‐reductases,^[2]^ alcohol dehydrogenases,^[5]^ Baeyer–Villiger monooxygenases,^[6]^ heme‐independent monooxygenases,^[7]^ P450 monooxygenases,^[8]^ and imine reductases^[9]^ with reaction rates as high as 150 units per gram cells.^[10]^ A major environmental advantage of cyanobacterial biotransformation is the sustainable production of the biocatalyst, with light as an energy source and CO_2_ as a carbon source.^[11]^ From a physiological viewpoint it is important to note that light‐driven biotransformations consume electrons from the photosynthetic electron transport chain, but do not require fixation of carbon dioxide. This could be an explanation why cyanobacterial photobiotransformations are much faster than the production of various C‐metabolites.^[12]^ For the metabolic engineering of cyanobacterial whole‐cell biocatalysts, both the variation of the intracellular enzyme concentration^[9]^ and the deletion of other electron‐consuming processes[^8b^, ^10^] have been proven as successful strategies. While photobiotransformations have been established with high reaction rates, the scale‐up of the reaction requires a photobioreactor geometry with very short light penetration pathways. On the one hand, this regards the overall light availability in a reactor, taking into account that the light penetration in water is limited and cells strongly absorb visible light. On the other hand, the reactor should have a continuous light distribution to avoid fluctuation, which has physiological consequences for the cells.^[13]^ Typical photobioreactors are based on flat‐panels or tube reactors with low‐to‐moderate surface/volume ratios (SVR). Reactor concepts with internal illumination can shorten light pathways.^[14]^ Among them is a new concept developed by Buchholz and co‐workers, which utilizes freely suspended wireless light emitters (WLEs).^[15]^ These WLEs consist of a single light emitting diode (LED) with a receiving coil encapsulated in a polycarbonate shell powered via induction by emitting coils from outside of the reactor. Among the various concepts mentioned above, this approach shows the highest SVR^[16]^ and provides evenly distributed light within the photobioreactors;^[15]^ this was exploited recently by Duong et al. for the intensification of a photoenzymatic reaction.^[17]^ This concept is especially intriguing as it allows to work with column reactors of a larger diameter than externally illuminated photobioreactors.

As a model system, we chose the light‐driven ene‐reduction of 2‐methylmaleimide (2‐MM) catalyzed by an ene‐reductase YqjM from *Bacillus subtilis* heterologously expressed in a model cyanobacterium *Synechocystis* sp. PCC 6803 (*Synechocystis* from here). This reaction proceeds with high stereoselectivity and represents a typical stereoselective redox reaction for the synthesis of fine chemicals. The specific activity of 180 μmol per hour and per mg chlorophyll a (μmol mg_chl*a*_^−1^ h^−1^)^[10]^ consumes a considerable part of the total photo‐production of nicotinamide adenine dinucleotide phosphate (NADPH) that has been estimated to be in the range of 530–1070 μmol mg_chl*a*_^−1^ h^−1^.^[18]^ In this article, we investigate the effect of internal illumination on the initial reaction rate and volumetric yield in the scale‐up of a photobiocatalytic asymmetric enzymatic ene‐reduction in recombinant cells of *Synechocystis*.

## Results and Discussion

**Figure 1:**
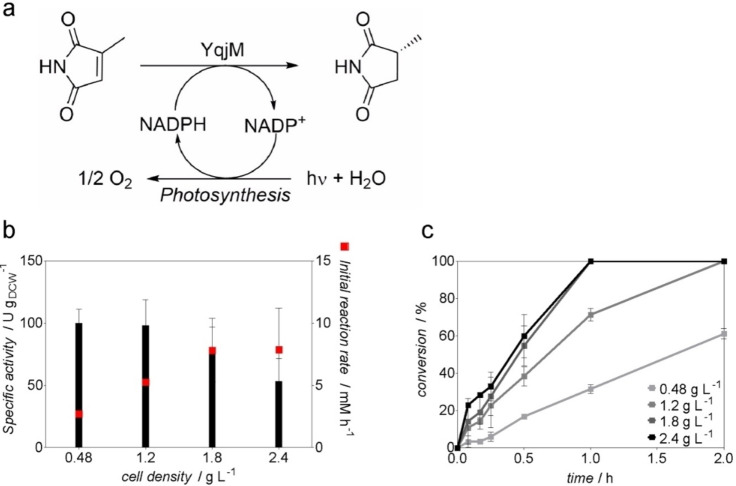
(a) Whole‐cell light‐driven biotransformations catalyzed by *Synechocystis* P_*cpcB*_::*yqjM*. (b) Specific activity and initial product formation rates achieved at 1 mL scale using various cell densities, independent biological triplicates. (c) Conversion of 2‐MM during photobiotransformations (1 mL, 10 mm 2‐methylmaleimide, 160 rpm, 30 °C, 200 μE m^−2^ s^−1^), independent biological triplicates.

*Synechocystis* P_*cpcB*_::*yqjM* was cultivated in 200 mL gas washing bottles under continuous light illumination reaching an optical density at 750 nm (OD_750_) of 1–3 (Supporting Information, Figure S3a), harvested and utilized in whole‐cell light‐driven reduction of 2‐MM to (*R*)‐2‐methylsuccinimide (2‐MS) (Figure 1a) with external illumination using LED lamps (200 μE m^−2^ s^−1^). As reported previously,[^2^, ^10^] the specific activity and the product formation rates showed a strong dependency on the cell density applied (Figure 1). Within the analyzed cell density range (0.48–2.4 g L^−1^) at 1 mL scale, the specific activity of the cells dropped notably at cell densities higher than 1.2 g L^−1^. Notably, the specific activity at 2.4 g L^−1^ was only about half of that at 0.48 g L^−1^ (Figure 1b). The correlation between the initial reaction rate with cell density was observed up to a cell density of 1.8 g L^−1^, where a further increase to 2.4 g L^−1^ did not improve the rate anymore.

**Figure 2:**
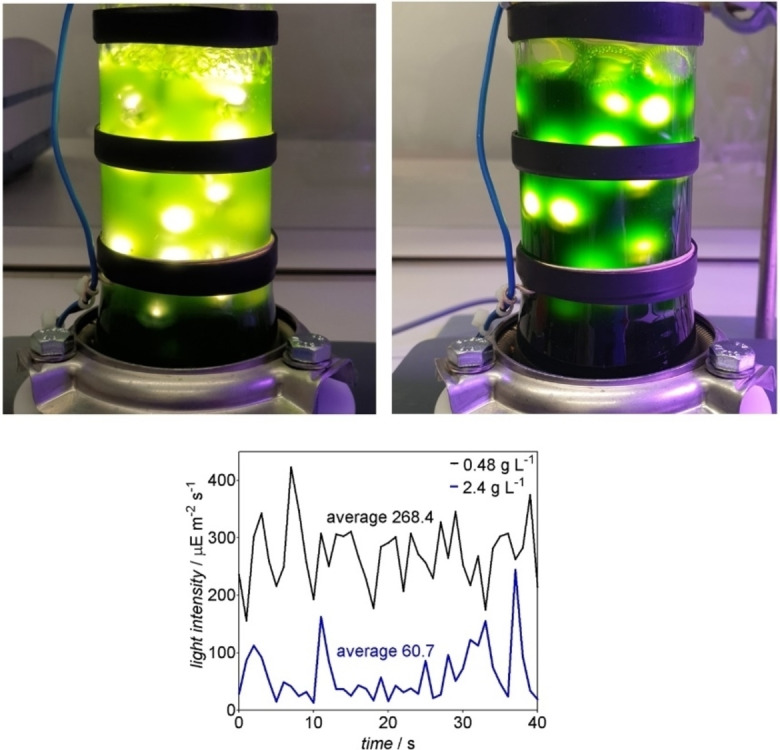
Top: Photo‐biotransformations performed in the BCR with internal illumination provided by 40 floating wireless light emitters at a cell density of 0.48 g L^−1^ (left side) and 2.4 g L^−1^ (right side). Bottom: determination of the light intensity inside BCR with internal illumination by 40 WLEs at a cell density of 0.48 or 2.4 g L^−1^, respectively.

The results in 1 mL scale show a clear decrease of productivity per cell at higher cell density. A larger reaction volume with longer light pathways is expected to increase this effect. Yet, achieving high reaction rates requires higher cell densities. Additionally, it should also be noted that a steady light intensity is important because fluctuating light affects the physiology of the cyanobacteria.^[19]^ On basis of these considerations, we reasoned that it would be difficult to achieve a high productivity at cell densities exceeding 1.8 g L^−1^ with external illumination. Internal illumination, however, provides much shorter light pathways and the beneficial effect for photo‐biocatalytic reactions has been demonstrated recently.^[2]^ Therefore, a bubble column reactor (BCR) with internal illumination provided by WLEs appeared to be the ideal solution for the intensification and scale‐up of cyanobacterial biotransformations (Figure 2).

**Figure 3:**
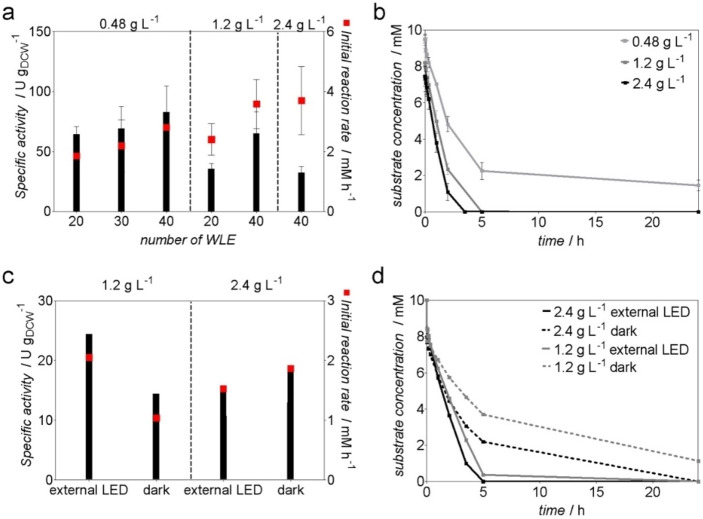
Whole‐cell biotransformation catalyzed by *Synechocystis* P_*cpcB*_::*yqjM* in a BCR. (a) Specific activity and initial product formation rate of *Synechocystis* P_*cpcB*_::*yqjM* in a BCR with internal illumination provided by WLEs (200 mL, 10 mm 2MM, 0.6 L min^−1^ airflow), independent biological triplicates. (b) Time course of the reactions using different cell densities with 40 WLEs, independent biological triplicates. (c) Specific activity and initial product formation rate of *Synechocystis* P_*cpcB*_::*yqjM* in a BCR externally illuminated with LED strips (300 μE m^−2^ s^−1^) or in dark (200 mL, 10 mm 2‐MM, 0.6 L min^−1^ airflow), single measurements. (d) Time course of the reactions with external illumination or in dark, single measurements.

The effect of the number of WLEs on the photo‐biotransformations was initially evaluated. Therefore, 20, 30, or 40 WLEs were used in a 200 mL reaction volume with *Synechocystis* P_*cpcB*_::*yqjM* at a cell density of 0.48 g L^−1^ and 30 °C and 10 mm 2‐methylmaleimide as a substrate. Among the investigated numbers of WLEs, the use of 40 yielded the highest specific activity of 83 U g_DCW_^−1^, which is well within the range of the rates observed in 1 mL‐scale experiments (Figure 3a and Figure 1b). At a cell density of 1.2 g L^−1^, 20 WLEs led to an activity of 35.8 U g_DCW_^−1^, whereas 40 WLEs allowed to reach an activity of 65.5 U g_DCW_^−1^ (corresponding to 5.31 U mg_Chla_^−1^) (Figure 3a). The initial product formation rate for the latter reaction was 3.6 mm h^−1^. Besides the overall availability of the light, its distribution should be as even as possible in the photobioreactor because the cells adapt to fluctuating light with the activation of protection systems such as flavodiiron proteins that constitute an additional electron sink.^[13]^ Figure 2 shows that at a cell density of 0.48 g L^−1^, the light distribution in the bubble column reactor is quite even. At a cell density of 2.4 g L^−1^, however, a considerable light fluctuation is apparent, both visually and by measuring at a specific site at different time points.

Further increasing the cell density to a value of 2.4 g L^−1^ resulted in shortening the reaction time, which allowed to achieve a full conversion after 3.5 h with an initial activity of 32.5 U g_DCW_^−1^ (2.74 U mg_Chla_^−1^) and an initial reaction rate of 3.7 mm h^−1^ (Figure 3a). At a cell density of 2.4 g L^−1^, the strong effect of the light absorption of the cells could be clearly seen as light intensity inside the BCR dropped by 53 % (Figure 2). Increasing the cell density from 0.48 to 2.4 g L^−1^ resulted in a 61 % decrease of the specific activity. This activity drop is stronger than the one observed in the externally illuminated 1 mL scale. Nevertheless, using internal illumination allowed to retain 80 % of activity at a cell density of 0.48 g L^−1^, 64 % at 1.2 g L^−1^, and 58 % at 2.4 g L^−1^ compared to the 1 mL scale reactions. Using cells at a density of 2.4 g L^−1^ resulted in the highest initial reaction rate of 3.7 mm h^−1^, corresponding to 47 % of the initial product formation rate from 1 mL scale. Keeping the reaction time short is not only important for cost‐effectiveness, but also needed for selectivity towards the target product as the cells can also unproductively consume the substrate 2‐MM (Supporting Information, Figure S6). Doubling the cell concentration and the number of WLEs increased the reaction rate almost two‐fold, as 1.2 g L^−1^ of cells with 20 WLEs resulted in 1.2 mm h^−1^ while 2.4 g L^−1^ and 40 WLEs resulted in 2.3 mm h^−1^.

Comparison of internal illumination using WLEs with external illumination and biotransformations without illumination underlined clearly the beneficial effect of the WLEs on the productivity of the whole‐cell biocatalyst. It is known that *Synechocystis* cells have the capacity to achieve some conversion in darkness or in presence of inhibitors of photosynthesis,[^2^, ^4g^, ^10^] which has been attributed to the utilization of storage compounds that were accumulated during the cultivation phase. During the light cycle, cyanobacteria produce NADPH via photosynthesis. In absence of light, the pentose phosphate pathway is assumed to be the main source of reducing equivalents.^[20]^ It should be noted, however, that the contribution of glycolytic routes in cyanobacteria is far from being understood.^[21]^ For these reasons, we expected some conversion for reactions in darkness or with external illumination. Indeed, external illumination with an intensity of 300 μE m^−2^ s^−1^ provided by LED strips (Supporting Information, Figure S7), resulted in a 75 % drop of specific activity for cell density of 1.2 g L^−1^ and 81 % for 2.4 g L^−1^.

At 1.2 and 2.4 g L^−1^ of cell density, the specific activity was 2.7 and 3‐fold higher, respectively, when using internal illumination with 40 WLEs, clearly showing the advantage of the WLE technology. The specific activities of *Synechocystis* P_*cpcB*_::*yqjM* at a cell density of 2.4 g L^−1^ externally illuminated were surprisingly similar to the reaction in darkness, although the dark reaction slows down over time (Figure 3d). The light measurements inside BCR using external illumination (2 μE m^−2^ s^−1^, Supporting Information, Figure S1c) show that cells might be facing darkness occurring locally or temporarily, which is a typical problem of large‐scale photobioreactors.^[16]^ Nevertheless, the comparison of the internal and external illumination clearly shows the advantage of the former for photobiotransformations at higher cell densities.

**Figure 4:**
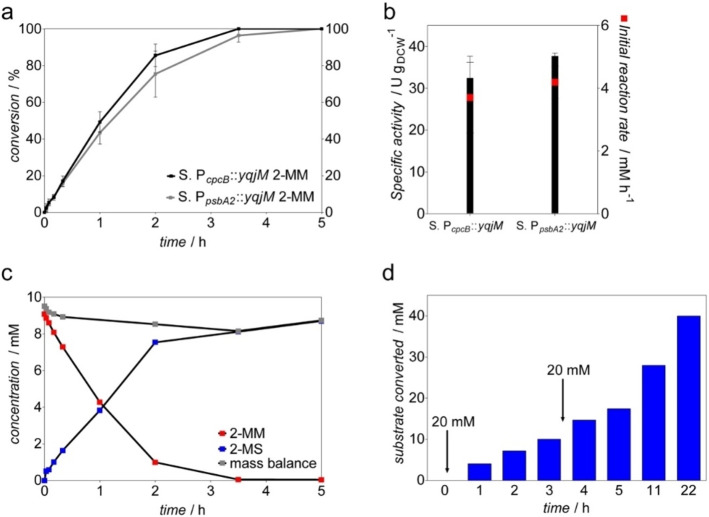
(a) Conversion of 10 mm 2‐MM into corresponding 2‐MS during photobiotransformations catalyzed by *Synechocystis* P_*cpcB*_::*yqjM* and *Synechocystis* P_*psbA2*_:*:yqjM* both at a cell density of 2.4 g L^−1^. (b) Specific activity and initial product formation rate of *Synechocystis* P_*cpcB*_::*yqjM* and *Synechocystis* P_*psbA2*_::*yqjM*. (c) Time course of the preparative 10 mm 2‐MM photobiotransformation catalyzed by *Synechocystis* P_*cpcB*_::*yqjM*, single measurements. (d) 2‐MM substrate converted by *Synechocystis* P_*cpcB*_::*yqjM* cells (2.4 g L^−1^) using a substrate feeding approach, with initial substrate concentration introduced 20 mm and a subsequent feeding of 20 mm at 3 h, single measurements.

We have shown previously that a higher cellular enzyme concentration leads to improved reaction rates.[^9^, ^10^] Therefore, we use the light‐inducible promoter P_*psbA2*_ for the expression of the enzyme. For induction, we cultivated the cells at a light intensity of 230 μE m^−2^ s^−1^, which is sufficient for an induction of this promoter. The results of the biotransformation with *Synechocystis* P_*psbA2*_::*yqjM* indicated a slight improvement in the initial specific activity of 38 U g_DCW_^−1^ compared to 32.5 U g_DCW_^−1^ obtained with *Synechocystis* P_*cpcB*_::*yqjM*, with 4.1 mm h^−1^ product formation rate (Figure 4b). In order to achieve fast full conversion, a reaction was run with *Synechocystis* P_*cpcB*_::*yqjM* at a cell density of 2.4 g L^−1^ and 40 WLEs using 10 mm 2‐MM as substrate (Figure 4c). The product was isolated after 5 h of the reaction. After cell removal and extraction with ethyl acetate, NMR analysis showed an isolated yield of 157.4 mg pure product (71 % yield) (*R*)‐2‐methylsuccinimide (2‐MS) in outstanding optical purity [99 % enantiomeric excess (*ee*)].

It should be noted that the product could be obtained in very high purity without the need for purification by flash chromatography, which simplifies the downstream processing and cuts the costs (Supporting Information, Figures S4 and S5).

Further intensifying the process by the addition of 40 mm 2‐MM by substrate feeding approach resulted in full conversion in 22 h (Figure 4d), producing 650 mg pure (*R*)‐2‐methylsuccinimide (73 % yield) with >99 % *ee*.

Previous light‐driven biotransformations scale‐up systems using stirred‐tank photobioreactors at the cell density of 0.5–0.8^[8a]^ or 1.6 g L^−1[22]^ point to the problem of high cell densities to be applied. Photobiotransformations could benefit from the developed technologies for the high cell density cultivation that reached up to 50 g L^−1^, such as thin‐layer cascade photobioreactors^[23]^ and CellDeg system cultivations.^[24]^ However, the application of these set‐ups in the scale‐up of light‐driven biocatalysis needs to be followed by the appropriate development of efficient light sources with beneficial light penetration depths, where WLEs and technologies such as light guides (e. g., optical‐fibers illumination^[25]^) can play an important role. The cost of the WLEs itself, their durability, and the efficiency of energy transfer via inductions might be drawbacks of the BCR; nevertheless, our results show that the principle of internal illumination bears clear advantages over external light supply. Future research will focus on the identification of the best way to combine an internal light source with an efficient mixing of the reaction, both in view of efficiency and practicability.

Easier handling and scalability of BCR with higher volume is an advantage of this technology, as these bioreactors can be filled up to higher volume without influencing SVR (while appropriately increasing the number of WLEs). This reactor concept is generally applicable for recently described whole‐cell biotransformations,[^1^, ^26^] keeping in mind that volatile substrates pose a problem. Other reactor concepts, such as capillary biofilm reactors operated at higher cell densities up to 52 g L^−1^, show another promising approach, but one of the bottlenecks for applications are the extreme long times for biofilm maturation (up to 5 weeks).^[27]^ One of the concerns with cultivation set‐ups for high cell density is the CO_2_ supply across the gas‐liquid interface, which could be problematic with longer BCR geometries. It should be noted, however, that the biotransformation does not require CO_2_ itself. Moreover, the presence of the ene‐reduction as a strong electron sink might alleviate the need of CO_2_ fixation from a metabolic point of view.^[28]^ While we indeed did not observe any positive effect of higher CO_2_ supply or NaHCO_3_ addition during small‐scale biotransformation reactions (unpublished), this idea remains to be proven experimentally. Another problem that might limit the applicable length of the BCR could be the accumulation of oxygen, which could lead to photoinhibition.^[29]^

## Conclusion

The strong light absorption of cyanobacteria is a main limiting factor for the scale‐up of cyanobacterial biotransformations. Here we demonstrate that internal illumination in a bubble column reactor can alleviate this limitation. At a cell density of 2.4 g L^−1^, internal illumination with 40 wireless light emitters in 200 mL reaction volume allowed to obtain a reaction rate of 3.7 mm h^−1^ and a specific activity of 32.5 U g_DCW_^−1^. An intensified reaction with stepwise feeding of 40 mm ran to completion: Isolation of 650 mg product (73 % yield) underlined the practical usefulness of the approach. The fact that purification of the compound was not necessary highlight the practical advantages of cyanobacterial biotransformation. Compared to external source of light, illumination by floating wireless light emitters allowed a more than two‐fold rate increase. The results demonstrate the principle of internal illumination as a means to overcome the intrinsic cell density limitation of cyanobacterial biotransformations, obtaining high reaction rates in a scalable photobioreactor.

## Experimental Section

### Chemicals

The substrate 2‐methylmaleimide (2‐MM) was synthesized as previously described.^[2]^ The product 2‐methylsuccinimide (2‐MS) was obtained as a white powder from Chiracon GmbH (Luckenwalde, Germany). All other chemicals were purchased from Sigma‐Aldrich (Steinheim, Germany) or Carl Roth (Karlsruhe, Germany) unless otherwise indicated.

### Strains

All *Synechocystis* strains utilized are listed in Table S1.

### Cultivation of *Synechocystis* strains

Seed cultures of *Synechocystis* were grown in liquid BG‐11 medium (pH 8) supplemented with 50 μg mL^−1^ kanamycin in 300 mL Erlenmeyer flasks with a working volume of 100 mL. The cultures were maintained in a plant growth chamber (SWGC‐1000, WISD lab instruments) fitted with white fluorescent lamps delivering a light intensity of 40–60 μE m^−2^ s^−1^. The cultures were placed on a rotary shaker (140 rpm) under ambient CO_2_ and 50 % humidity and a temperature of 30 °C. Cell growth was monitored by measuring the OD_750_. Upon reaching OD_750_=1–2, the cells were harvested by centrifugation (24 °C, 15 min, 3500 rpm), resuspended in fresh BG‐11, and inoculated in gas washing tubes (*V*=200 mL) to OD_750_=0.1. The tubes were maintained in an aquarium regulated at 30 °C and illuminated with six fluorescent lamps delivering a light intensity of 200–250 μE m^−2^ s^−1^ (Supporting Information, Figure S3b). Aeration and mixing of the cultures were ensured by bubbling using an air pump (Boyu S‐4000B pump) and filtered through a 0.2 μm filter to ensure the sterility. 

### Whole‐cell biotransformation

Upon reaching an OD_750_=1–3, cells were harvested by centrifugation (24 °C, 15 min, 3500 rpm). The pellet was resuspended in an appropriate volume of BG‐11 to a specific OD_750_ and subsequently utilized in whole‐cell biotransformations 10 mm 2‐MM with a working volume of 200 mL (stock solution in BG‐11). OD_750_=10 corresponded to 2.4 g L^−1^ of cell density, as previously determined.^[10]^ Reactions were performed using a bubble column reactor (BCR; *φ*_I_=5 cm, *h*=50 cm) with a working volume of 200–800 mL fitted with emitting coils. WLEs in varying numbers were suspended in the reactor to provide internal illumination. The WLEs consist of a white LED plus a receiving coil inside a polycarbonate shell (Supporting Information, Figure S2b). Air was supplied by a pump (Boyu S‐4000B) at a rate of 0.6 L min^−1^. A fan was utilized to provide cooling for reactions to keep the temperature at 30 °C. Reaction temperature was measured four times over the course of 1 h, then again after 3.5 and 20 h, and was found to be stable and did not increase above 30 °C. Large‐scale biotransformations with external illumination were performed by wrapping the BCR with LED stripes (BOXXX) (Supporting Information, Figure S7). The light intensity was maintained constant as an internally illuminated reactor.

For small‐scale biotransformation, the cells obtained from centrifugation were resuspended in fresh BG‐11 to OD750=20 and adjusted accordingly. Reactions were performed in 5 mL glass vials with a working volume of 1 mL and initiated with the addition of 10 mm 2‐MM (stock solution: 100 mm in BG‐11). The vials were placed in a bioreactor maintained at 30 °C (160 rpm) and equipped with LED lamps with an intensity of 200 μE m^−2^ s^−1^. Aliquots (100 μL) of the reaction mixture were taken at several time points (0, 2, 5, 10, and 20 min, 1, 2, 3.5, 5, and 24 h) and immediately frozen in liquid nitrogen. Samples were stored at −20 °C prior to GC analysis. Specific activities were calculated at <10 % substrate conversion.

### GC analysis

The substrate 2‐MM and its corresponding product 2‐MS were analyzed using a GC equipped with a flame‐ionization detector (FID) (GC‐2010 Plus, Shimadzu, Japan) outfitted with a ZB‐5 column. Samples (100 μL) were extracted with ethyl acetate containing 2 mm *n*‐decanol as an internal standard. The organic phase was dried in anhydrous MgSO_4_ and subsequently analyzed. The *ee* of the obtained product after biotransformation was analyzed using chiral GC‐FID (GC‐2030, Shimadzu, Japan) equipped with a β‐6ΤΒDAc column. Detailed GC procedure is given in Table S2.

### Determination of light intensity

The light intensity was measured using an LI‐250A light meter (LICOR Biosciences, Hamburg, Germany) equipped with a spherical micro quantum sensor US‐SQS/L (Walz, Effeltrich, Germany). Values were taken as an average (15 s) as well as a plot for 40 s (Supporting Information, Figure S2a).

**Product isolation**

After the reaction, the mixture was extracted three times with ethyl acetate (1 : 1.33, *v*/*v*). The organic layer was dried in anhydrous MgSO_4_ and evaporated in vacuo. The obtained product was analyzed using chiral GC‐FID and ^1^H and ^13^C NMR spectroscopy.

### Quantification of chlorophyll a content

The amount of chlorophyll a was determined as previously described.^[10]^ A sample of cell culture (100 μL) with a known OD_750_ was pelleted, resuspended in 100 μL dH_2_O, and 900 μL methanol was added. Samples were briefly vortexed and incubated in darkness for 10 min. The absorption at 665 nm was measured and the amount of chlorophyll a was determined using the extinction coefficient *ϵ*=78.74 L g^−1^ cm^−1^.

## Conflict of interest

The authors declare no conflict of interest.

## Supporting information

As a service to our authors and readers, this journal provides supporting information supplied by the authors. Such materials are peer reviewed and may be re‐organized for online delivery, but are not copy‐edited or typeset. Technical support issues arising from supporting information (other than missing files) should be addressed to the authors.

Supporting Information
