# Supplementary material for: Internal Illumination to Overcome the Cell Density Limitation in the Scale‐up of Whole‐Cell Photobiocatalysis
Source: ChemSusChem. 2021 Jul 6;14(15):3219–25. doi: 10.1002/cssc.202100832 (PMC8456840; doi:10.1002/cssc.202100832)
Supplement: Supplementary file 1 — Supporting Information [file CSSC-14-3219-s001.pdf]

ChemSusChem

Supporting Information

Internal Illumination to Overcome the Cell Density Limitation in the Scale-up of Whole-Cell Photobiocatalysis

Markus Hobisch[+], Jelena Spasic[+], Lenny Malihan-Yap, Giovanni Davide Barone, Kathrin Castiglione, Paula Tamagnini, Selin Kara,* and Robert Kourist*© 2021 The Authors. ChemSusChem published by Wiley-VCH GmbH. This is an open access article under the terms of the Creative Commons Attribution License, which permits use, distribution and reproduction in any medium, provided the original work is properly cited.

Contents

List of Abbreviations 3

Supporting Tables and Figures..4

Table S1. Strains utilized in this work. 4

Table S2. Gas Chromatography Flame Ionization Detector Method...4

Figure S1. Light Intensity Measurements inside BCR...6

Figure S2. a) Schematic illustration of the Bubble Column Reactor...7

Table S3. Average light intensity in BCR 7

Figure S3. a) Cultivation of *Synechocystis* in gas washing tubes in an aquarium..........................8

Figure S4. ^{1}H NMR of 2-MS obtained after whole-cell biotransformation.......................................10

Figure S5. ^{13}C NMR of 2-MS obtained after whole-cell biotransformation......................................10

Figure S6. Consumption of 2-MM substrate over time by *Synechocystis* sp. PCC 6803 WT..............11

Figure S7. External illumination of BCR provided by LED strips..11

Figure S8. Example GC-Chromatogram 12

Figure S9. Emission spectrum of a white WLE. 12

References...13

List of Abbreviations

In alphabetical order

**2-MM**	2-Methylmaleimide
**2-MS**	2-Methylsuccinimide
**BCR**	Bubble Column Reactor
**chl*a***	Chlorophyll *a*
**DCW**	Dry Cell Weight
**GC-FID**	Gas Chromatography Flame Ionization Detector
**LED**	Light Emitting Diode
**NADPH**	Nicotinamide Adenine Dinucleotide Phosphate
**NMR**	Nuclear Magnetic Resonance
**OD_{750}**	Optical Density at 750 nm
**PAM**	Pulse Amplitude Modulation
Synechocystis	*Synechocystis sp.* PCC 6803
**WLE**	Wireless Light Emitters
**WT**	Wild Type

Supporting Tables and Figures

Table S1. Strains utilized in this work.

Strains	Description	Reference
Synechocystis sp. PCC 6803 WT	Wild type cyanobacterium *Synechocystis sp.* PCC 6803	[1]
Synechocystis sp. PCC 6803 P_{cpcB}::*yqjM*	Transgenic *Synechocystis sp.* PCC 6803 constructed in WT background harboring the gene *yqjM* from *Bacillus subtilis* under the control of the P_{cpcB} promoter in the genome locus *slr0168*	[2]
Synechocystis sp. PCC 6803 P_{psbA2}::*yqjM*	Transgenic *Synechocystis sp.* PCC 6803 constructed in WT background harboring the gene *yqjM* from *Bacillus subtilis* under the control of the promoter P_{psbA2} in the genome locus *slr0168*	[2]

Table S2. Gas Chromatography Flame Ionization Detector Method.

Achiral GC-FID (GC-2010 Plus, Shimadzu, Japan)

**Column**	Phase	ZB-5 (5% phenyl, 95% dimethyl polysiloxane)
	Dimension	Length = 30 m; Film Thickness = 0.25 µm; Inner Diameter = 0.32 mm
	Temperature	100 °C
**FID Detector**	Temperature	320 °C
	Air flowrate	400 mL min^{-1}
	H_2 flowrate	40 mL min^{-1}
**Injection port**	Volume	1.0 µL
	Carrier Gas	N_2 (1.5 mL min^{-1})
	Temperature	230 °C
	Split Ratio	20
**Temperature**	Program	1) 80 °C for 1 min; 2) 80-160 °C (50 °C min^{-1}); 3) 160-220 (15 °C min^{-1}); 4) 220-300 °C (50 °C min^{-1}) and 5) 300 °C for 3.8 min

The percent enantiomeric excess (% *ee*) was determined using Chiral GC-FID as detailed below:

Chiral GC-FID (GC-2030 Plus, Shimadzu, Japan)

**Column**	Phase	β-6TBDAc (Column ID: 23254-3)
	Dimension	Length = 50 m; Film Thickness = 0.25 μm; Inner Diameter = 0.25 mm
	Temperature	180 °C
**FID Detector**	Temperature	250 °C
	Air flowrate	200 mL min^{-1}
	H_2 flowrate	32 mL min^{-1}
**Injection port**	Volume	1.0 μL
	Carrier Gas	N_2 (24 mL min^{-1})
	Temperature	230
	Split Ratio	100
**Temperature**	Program	1) 180 °C (2 min); 2) 220 °C (5 °C min^{-1} for 3min)

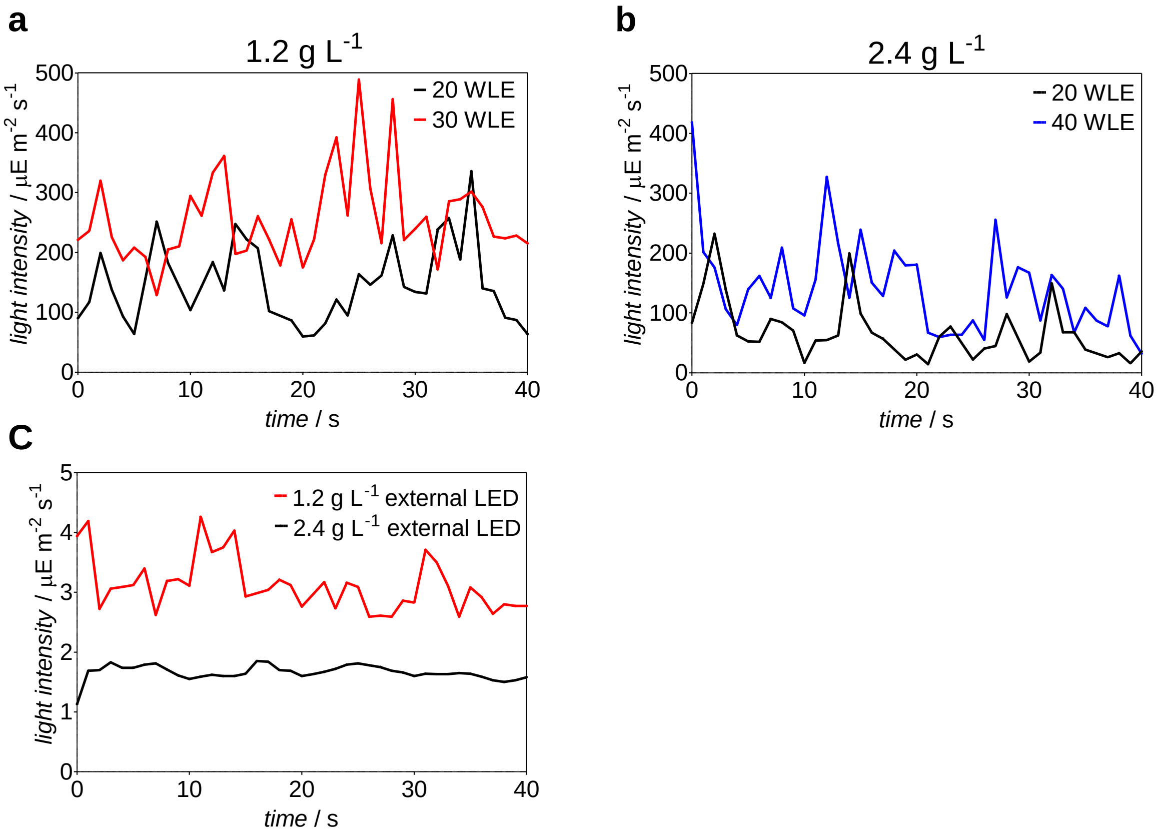

Figure S1. Light Intensity Measurements inside BCR with a) internal illumination at cell density 1.2 g L^{-1}; b) internal illumination at cell density 2.4 g L^{-1}; c) external illumination provided by LED strips.

The light intensity of the cyanobacterial mixture was determined using an LI-250A light meter (LI-COR Biosciences UK Ltd, Cambridge, United Kingdom) with a US-SQS/L light sensor (Waltz, Effeltrich, Germany) submerged at a depth of 5 cm from the surface in the center of the reactor.

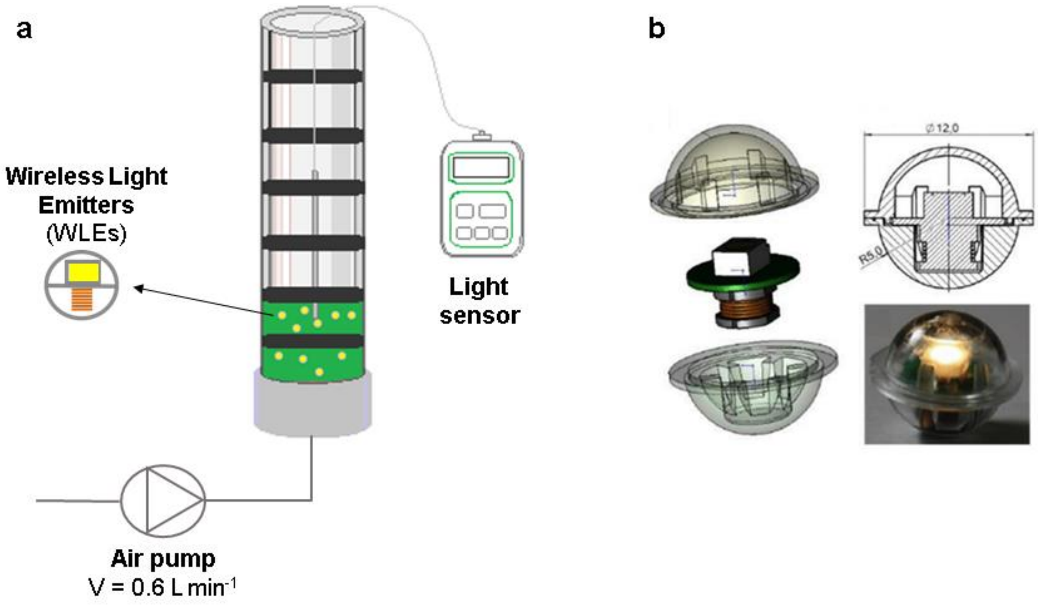

Figure S2. a) Schematic illustration of the Bubble Column Reactor with a working volume of 200 mL cyanobacterial mixture, air was delivered using a pump at a flowrate of 0.6 L min^{-1}; b) Wireless Light Emitters (WLEs) utilized for internal illumination in the BCR.[3]

Table S3. Average light intensity in BCR

**Conditions**	**Average light intensity [µE m^{-2} s^{-1}]**
BG11 WLE 40	302.3
BG11 external LED stripes	300.5
0.48 g L^{-1} WLE 20	145.1
0.48 g L^{-1} WLE 30	238.1
0.48 g L^{-1} WLE 40	268.4
1.2 g L^{-1} WLE 20	76.3
1.2 g L^{-1} WLE 40	142.3
2.4 g L^{-1} WLE 40	60.7
1.2 g L^{-1} external LED stripes	3.01
2.4 g L^{-1} external LED stripes	1.66

Synechocystis was maintained in an aquarium-type reactor at 30 °C using a heater (Thermo Fisher Scientific, Waltham, USA). Each gas washing tube contains 200 mL of cell culture supplemented with 50 µg mL^{-1} of kanamycin as a selectable marker. The cells were allowed to grow to an OD_{750} of 1-3, harvested, centrifuged (24 °C, 15min, 3500 rpm) and subsequently

utilized in whole-cell light driven biotransformations. The OD_{750} 10 corresponded to 2.4 g L^{-1} of cell density as previously determined.[2] For this cell density, measured chlorophyll a content was in the range of 29-31 μg mL^{-1}.

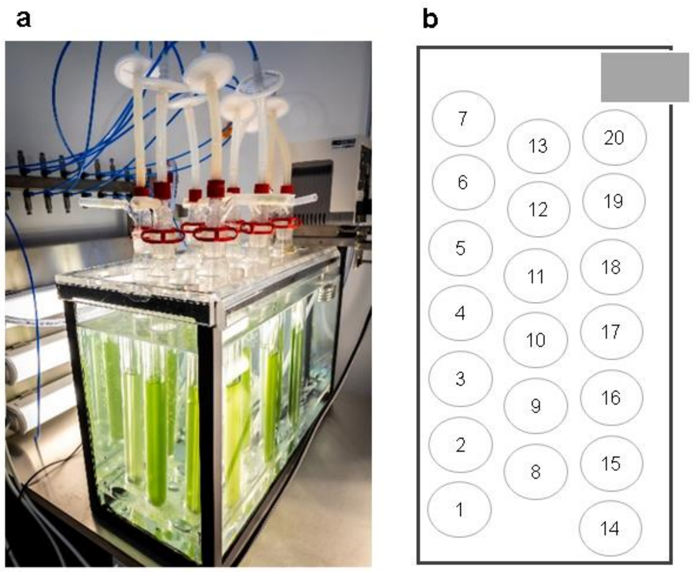

Slot #	Light Intensity	Slot #	Light Intensity
1	207.8	11	243.1
2	230.6	12	232.4
3	249.9	13	211.9
4	256.1	14	220.0
5	250.3	15	222.7
6	239.6	16	227.0
7	220.4	17	223.5
8	218.7	18	220.7
9	232.7	19	208.8
10	240.0	20	197.48

Light Intensity in μE m^{-2} s^{-1}

Figure S3. a) Cultivation of *Synechocystis* in gas washing tubes in an aquarium maintained at 30 °C, air was delivered using a pump and passed through 0.2 μm filters; b) Light intensity measurements for each tube (measured in water).

Synthesis of 2-Methylmaleimide

2-Methylmaleimide was synthesized as described elsewhere.[4] In a typical reaction, citranoic anhydride (89 mmol) and ammonium acetate (178 mmol) were reacted in acetic acid (40 mL). The mixture was allowed to heat under reflux for 2.5 h. After the reaction, the solvent was partially removed in vacuo. The remaining solution was mixed with 100 mL of saturated NaCl and 40 mL of water. The solution was then extracted with ethyl acetate (8 x 25 mL). The crude product was purified by silica column chromatography using a mobile phase of 5:1 (Cyclohexane: Ethyl acetate) obtaining a white solid product (3.5 g, 35.3% yield).

^{1}H NMR (300 MHz, DMSO-*d6*) δ 10.73 (s, br, 1H, NH), δ 6.54-6.56 (m, 1H, =CH-), δ 1.94 (J_{HH}=1.5 Hz, d, 3H, $CH_{3,}$)

^{13}C NMR (300 MHz, $CDCl_3$) δ 10.377, δ 128.166, δ 146.129, δ 172.422, δ 173.310

2-MS Extraction and characterization

Optically pure (> 99% ee) 2-MS was isolated as a solid powder (157.4 mg, 71% yield). The product was analyzed by ^{1}H and ^{13}C NMR.

^{1}H NMR (300 MHz, $CDCl_3$) δ 7.86 (s, br, 1H, NH), δ 3.02–2.81 (m, 2H, –CH_2-), δ 2.46-2.23 (m, 1H, -CH-), δ 1.41–1.28 (m, 3H, –CH_3)

^{13}C NMR (300 MHz, $CDCl_3$) δ 17.098, δ 36.768, δ 38.194, δ 176.618, δ 180.920

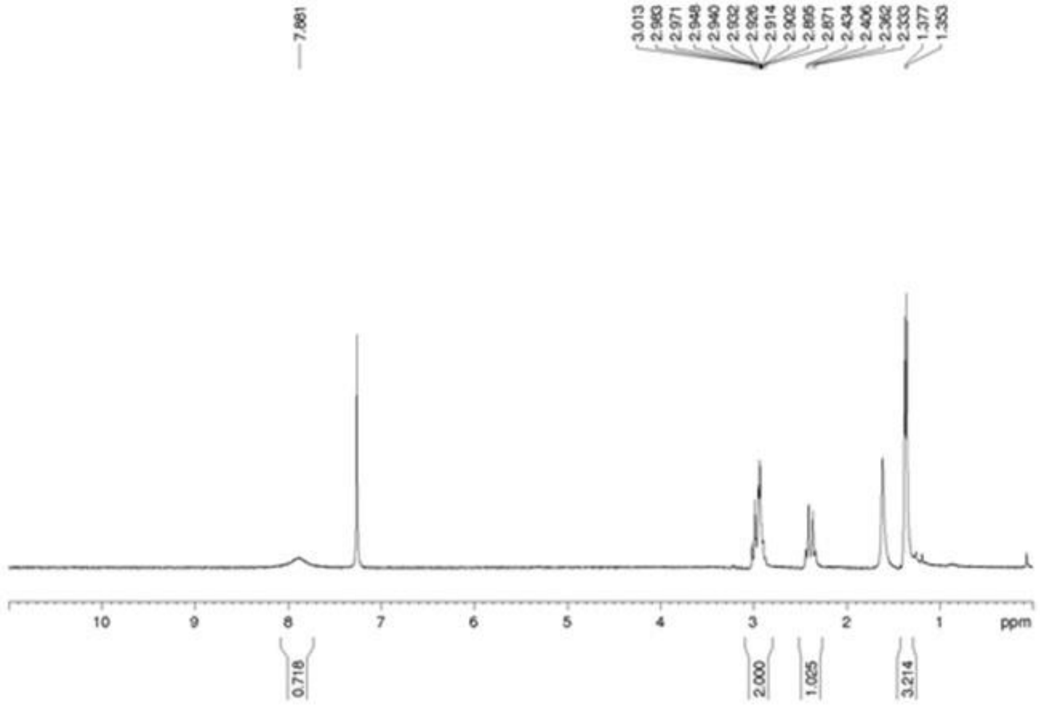

Figure S4. ^{1}H NMR of 2-MS obtained after whole-cell biotransformation.

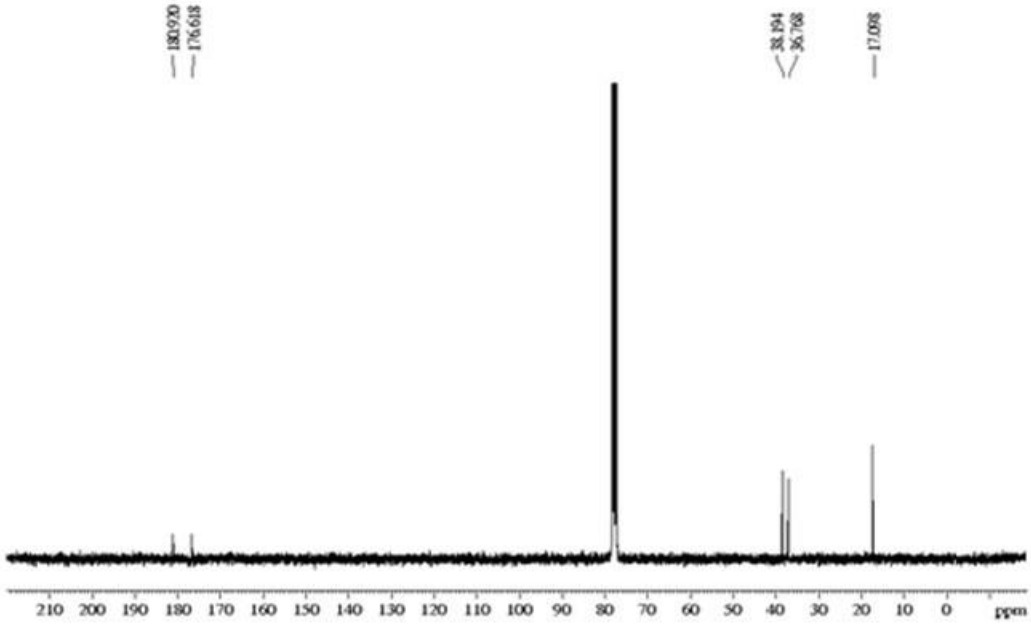

Figure S5. ^{13}C NMR of 2-MS obtained after whole-cell biotransformation.

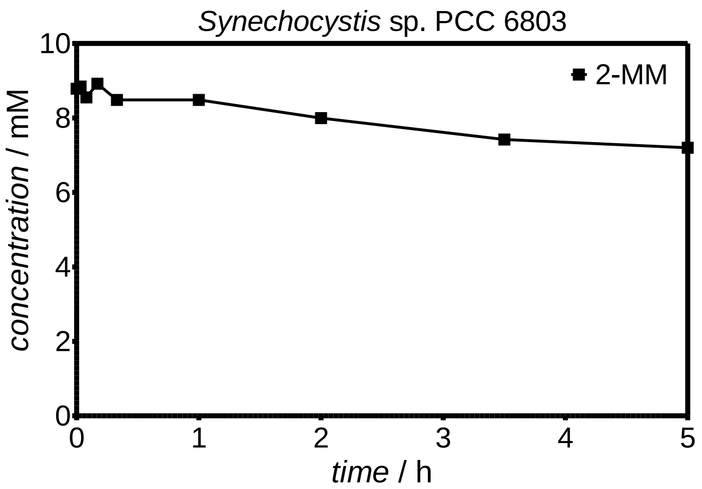

Figure S6. Consumption of 2-MM substrate over time by *Synechocystis* sp. PCC 6803 WT (1.2 g L^{-1}, 200 mL, 10 mM 2-methylmaleimide, 0.6 L min^{-1} airflow, 40 WLEs).

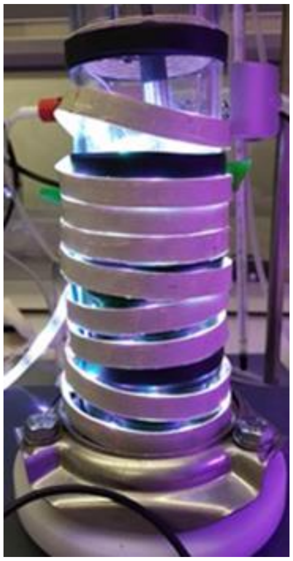

Figure S7. External illumination of BCR provided by LED strips (BOXXX)

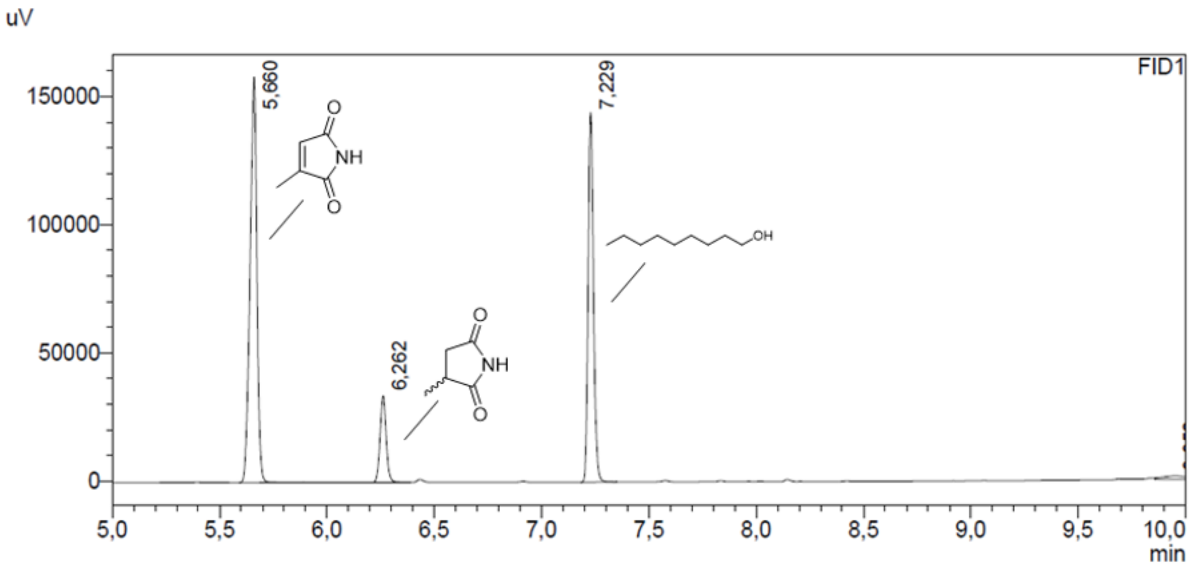

Figure S8. Example GC-Chromatogram with 2-MM substrate (ret. time 5.660 min) and 2-MS product (ret. time 6.262 min). *n*-decanol (ret. time 7.229 min) was used as an internal standard.

Measurement of emission spectrum

The emission spectrum of a white WLE was measured with a MSC15 spectrometer (Gigahertz-Optik, Türkenfeld, Germany).

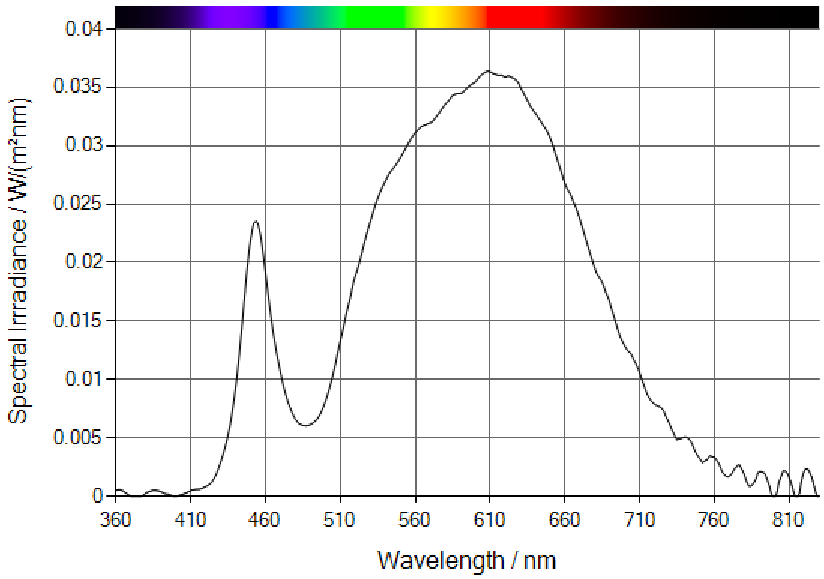

Figure S9. Emission spectrum of a white WLE.

References

[1] R. Y. Stanier, R. Kunisawa, M. Mandel, G. Cohen-Bazire, *Bacteriol. Rev.* **1971**, *35*, 171–205.

[2] L. Assil-Companioni, H. C. Buchsenschutz, D. Solymosi, N. G. Dyczmons-Nowaczyk, K. K. F. Bauer, S. Wallner, P. Macheroux, Y. Allahverdiyeva, M. M. Nowaczyk, R. Kourist, *ACS Catal.* **2020**, *10*, 11864-11877.

[3] M. Heining, R. Buchholz, *Biotechnol. J.* **2015**, *10*, 1131-1137.

[4] K. Koninger, A. Gomez Baraibar, C. Mugge, C. E. Paul, F. Hollmann, M. M. Nowaczyk, R. Kourist, *Angew. Chem. Int. Ed.* **2016**, *55*, 5582-5585; *Angew. Chem.* **2016**, *128*, 5672-5675.
